# Tuberculosis: Epidemiology and Control

**DOI:** 10.4084/MJHID.2014.070

**Published:** 2014-11-01

**Authors:** Giorgia Sulis, Alberto Roggi, Alberto Matteelli, Mario C. Raviglione

**Affiliations:** 1 University Division of Infectious and Tropical Diseases, WHO Collaborating Centre for TB/HIV collaborative activities and for TB elimination, University of Brescia, Brescia, Italy; 2 Global Tuberculosis Programme, World Health Organization, Geneva, Switzerland

## Abstract

Tuberculosis (TB) is a major public health concern worldwide: despite a regular, although slow, decline in incidence over the last decade, as many as 8.6 million new cases and 1.3 million deaths were estimated to have occurred in 2012. TB is by all means a poverty-related disease, mainly affecting the most vulnerable populations in the poorest countries. The presence of multidrug-resistant strains of *M. tuberculosis* in most countries, with somewhere prevalence is high, is among the major challenges for TB control, which may hinder recent achievements especially in some settings. Early TB case detection especially in resource-constrained settings and in marginalized groups remains a challenge, and about 3 million people are estimated to remain undiagnosed or not notified and untreated. The World Health Organization (WHO) has recently launched a new global TB strategy for the “post-2015 era” aimed at “ending the global TB epidemic” by 2035. This strategy is based on the three pillars that emphasize patient-centred TB care and prevention, bold policies and supportive systems, and intensified research and innovation. This paper aims to provide an overview of the global TB epidemiology as well as of the main challenges that must be faced to eliminate the disease as a public health problem everywhere.

## Introduction

Tuberculosis (TB) is one of the major public health threats, competing with the human immunodeficiency virus (HIV) as the cause of death due to infectious diseases worldwide. Although a declining trend in TB incidence, prevalence and mortality has been observed over the last decade, elimination of the disease at global level is still out of reach, and massive resource investment is still required. TB is a poverty-related disease which disproportionately affects the poorest, the most vulnerable and marginalized population groups wherever it occurs. Improving access to diagnosis and care, the basic requirements in the fight against TB, are particularly challenging in these persons. Besides, TB control cannot be carried out without setting up an effective surveillance system in order to define the course of the epidemic and assess the impact of control measures on the disease. Hence, TB national programs must devote significant resources to the disease-specific recording and reporting system. Routine surveillance systems represent the best method for drug resistance assessment and monitoring, though high-quality data can be generated only by the allocation of significant resources. The increasing number of detected multidrug-resistant forms is among the current most frightening issues, requiring a strong and comprehensive commitment in terms of funds allocation, research promotion and field implementation of new tools and protocols.

## Global Epidemiology

According to the World Health Organization (WHO), about 8.6 million cases (8.3–9.0 million) were estimated to have occurred in 2012, approximately 2.9 of whom were in women. Most cases are estimated to be in Asia and Africa (58% and 27% respectively), with the highest incidence in India (range 2.0–2.4 million) and China (0.9 −1.1 million), together accounting for 38% of the total number of cases.^1^

**Figure 1:**
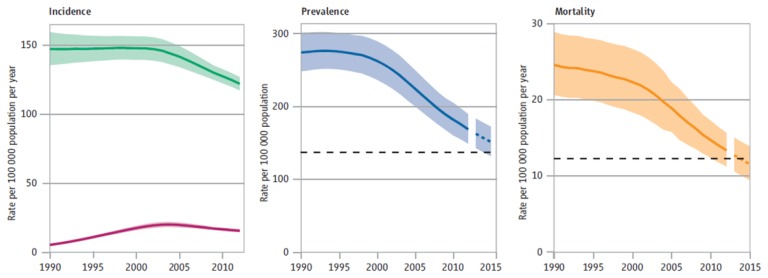
Global trends in estimated rates of TB incidence, prevalence and mortality 1990–2012 and forecast TB prevalence and mortality rates 2013–2015 [*World Health Organization (WHO), Global Tuberculosis Report 2013*].

The global TB incidence rate slowly declined from 1997 to 2001, with an increase in 2001 (due to the rising number of cases among HIV-infected patients in Africa) (Figure 1). Subsequently, a 1.3% per year average reduction rate has been observed since 2002, reaching 2.2% between 2010 and 2011. The absolute number of cases is also currently decreasing, though this declining trend only began in 2006. Based on these findings, the Millennium Development Goal 6 Target for tuberculosis (i.e. “to halt and begin to reverse the incidence”) has already been achieved.

Twelve million (11–13 million) prevalent cases of TB were estimated in 2012, corresponding to about 169 cases per 100 000 population.^1^ TB prevalence is declining globally since the early 1990s (before incidence started to decline). This decline is largely attributed to the progressive introduction of the DOTS strategy which, by emphasizing bacteriological diagnosis and standard short-course chemotherapy with direct observation of treatment, may have significantly contributed to the reduction of chronic and untreated cases, as well as to the duration of illness (Figure 1). Nevertheless, the Stop TB Partnership target of halving the 1990 prevalence rate by 2015 will probably be missed (a reduction of 37% was registered in 2012, 169 / 100 000 compared to 263 / 100 000 in 1990), because of the delays in the African and the European WHO regions.

TB mortality was estimated at 1.3 million deaths (1.0–1.6 million) in 2012, including 320 000 (300 000 – 340 000) HIV-associated cases. A 45% drop in TB mortality rate has been observed globally since 1990 (Figure 1).^1^

The traditional case detection rate (CDR), defined as the proportion of notified cases among the estimated number of new and relapse TB cases, thought to have occurred in a given year, is a problematic indicator in TB epidemiology, though it could potentially provide very useful information on the “diagnostic capacity” of a TB control program.^2^ The denominator consists of an estimate: significant efforts are currently ongoing to obtain reliable estimates through the performance of costly prevalence surveys but coverage is still limited.^3^ In 2012, 6.1 million TB cases were notified by the National TB Programs (NTPs). 5.4 million were new cases, and 0.3 million were relapses (with India and China showing the highest notification rates: 39% overall); 0.4 million cases of retreatment (excluding relapses) were also reported. Most newly diagnosed patients had pulmonary TB, and more than half of them were sputum smear positive.^1^ CDR reached 66% (64–69%) in 2012 with several regional differences. In other words, one-third of cases, corresponding to an estimated 3 million cases, was missed that year. This implies that a significant proportion of TB patients remains either unrecognized and untreated or not notified. The former actively contribute to further transmission of the disease. The latter may be detected outside of national programs and managed inappropriately, also contributing to further transmission and creation of drug resistance. Further improvements in diagnostic capacity and surveillance system are needed in some Regions such as South-East Asia, Africa, and Eastern Mediterranean.

Treatment outcome represents a useful process indicator to be closely monitored, being a measure of progress in expanding access to quality-assured care. Approximately 22 million lives are estimated to have been saved since 1995, when the DOTS strategy was introduced. The proportion of successfully treated patients, currently reaching 87% at global level, is significantly lower than the average, in some WHO regions, like the European one (probably due to the high failure rate associated to MDR-TB) and in the African one (due to the high rate of deaths or defaulting linked to HIV co-infection).^1^

## The Challenge of Controlling the Disease in the most Vulnerable Populations

Since TB does not homogeneously affect the population, selected high-risk groups should be identified in all settings as they deserve special attention and should be addressed specifically with additional interventions.

TB is mostly a poverty-related disease: this can explain its uneven distribution in different population groups. Poor housing and environmental conditions, food insecurity, financial difficulties, illiteracy, unfavourable psycho-social circumstances are among the major determinants of TB and concomitantly affect the capacity of sick persons to access healthcare services.^4^–^9^

Well-defined vulnerable groups include people living with HIV infection, prisoners, homeless people, migrants/refugees, and substance or heavy alcohol users. Besides the increased risk of exposure to *M. tuberculosis*, vulnerable groups are also more likely to progress to active disease once they are infected due to the immunocompromised status of their underlying condition. Moreover, in some of these groups TB may remain for a long time undiagnosed, thus representing a source of infection for the entire community.^10^

Social marginalization is often responsible for a limited access to health services leading to diagnostic delay, clinical worsening and poor adherence to treatment, and eventually to a less favourable outcome.^11^–^13^ A common discouraging factor to seek medical care is the fear of stigmatization that is also an important determinant of poor adherence. The most fragile populations should be identified in each country in order to develop and implement tailored interventions aimed at addressing the needs of hard-to-reach groups. Global disease control will not be achieved without a cross-cutting approach towards these social determinants of the disease.

### TB/HIV co-infected patients

People living with HIV/AIDS (PLHIV) are at extremely high risk of TB, due to the immunological impairment associated to this infection and to frequent co-existence of deprived social conditions.

In 2012, 1.1 million TB cases (i.e. 13% of the total) were estimated to have occurred among PLHIV, whom 75% were reported in a few African countries; where more than 60% of tested TB patients resulted HIV-positive. Notably, since 2.8 million notified TB patients knew their HIV status, and 20% of those tested were positive, the number of notified HIV-infected TB patients was approximately 550 000. This group represents about half of the number of cases estimated to have occurred that year.

The number of deaths among HIV-infected TB patients amounted to 0.3 million in 2012 with no relevant differences between men and women.

Data from countries that reported treatment outcomes disaggregated by HIV status showed a lower treatment success rate for new HIV-positive TB cases (73%) compared to HIV-uninfected cases (87%).^1^ The case fatality rate is specifically high among HIV-infected TB patients: merging the death and default outcomes (most defaulting cases will eventually die), this category accounts for 19% of HIV-positive treated cases globally.

All newly diagnosed TB patients should be aware of their HIV condition, according to recommended collaborative TB/HIV interventions.^14^ The trend in the percentage of TB patients knowing their HIV status has continued to increase since 2004, reaching a peak of 46% in 2012.^1^ However, HIV testing coverage varied widely between countries, with a 74% peak in the African region. Much lower rates were reported in the Eastern Mediterranean Region (EMR) and South-East Asia Region (SEAR) (14% and 39% respectively), while the European (EUR) and American Regions (AMR) slightly exceeded a 60% coverage.^15^

Though WHO recommends that all TB/HIV co-infected patients must receive timely antiretroviral therapy (ART) regardless of their CD4+ count,^16^ this goal is still far from being achieved satisfactorily in most countries with an average coverage of 57% in 2012 at global level (55% in the African region). Expanding the coverage of ART among HIV-infected TB patients represents the highest current priority for TB/HIV interventions in countries with a high burden of both diseases.

Furthermore, data on the provision of isoniazid preventive therapy (IPT) to PLHIV show an unsatisfactory level of implementation. Only 42 countries currently reported on this indicator and just 0.5 million persons initiated it in 2012. In short, indicators to monitor TB/HIV interventions suggest that progress has been real in the past decade. At the same time, some interventions with life-saving potential such as timely ARV administration and IPT are not yet fully implemented.

### TB in prisons

The world prison population is currently estimated at approximately 8–10 million people with a considerably high turnover.^17^ TB represents a major concern for jailed persons, due to overcrowding, inadequate nutrition and unsuitable medical services.^18^–^20^ However, the recognition of the magnitude of TB in prison remains a challenge due to weaknesses of the information system in the penitentiary system. In a systematic review of the literature, the median incidence rate ratio for tuberculosis in inmates compared to the general population was 23. 21 Exposure in prison accounted for a substantial population attributable fraction of tuberculosis in diverse geographical settings.^21^ It is estimated that up to 25% of a country’s TB burden may be due to the cases occurring among detainees.^17^ Prison inmates often share narrow spaces with insufficient air circulation which constitute the ideal conditions for transmission of *M. tuberculosis* via airborne droplets. Furthermore, social degradation is often already present at the time of incarceration, since many prisoners belong to marginalized groups (e.g. drug addicts, alcohol abusers, homeless people, migrants) and bear an even higher risk burden in such a confined environment. Besides the exposure to several risk factors for infection acquisition and disease progression, the penitentiary system is often unable to provide adequate TB screening services at first admission, with delayed application of isolation measures and increased time-to-treatment. Furthermore, care discontinuation after release is another relevant issue.^22^ In fact, casual and interrupted treatment significantly contributes to the development and spread of drug-resistance, creating a TB reservoir which threatens the entire community through internal personnel, visitors and former inmates.^23^

### TB in migrant populations

Migration is a common and complex phenomenon which has a profound influence on population dynamics and the global epidemiology of diseases. Most of the estimated 232 million international migrants, that currently cross the borders throughout the world annually (plus an additional 700–800 million internally displaced persons), originate from low-income countries, where the largest proportion of the global TB burden concentrates.^24^

Like many other airborne infections, TB has no boundaries and easily spreads from one region to another, just following people movements. Migrants represent the major reservoir of infection in many low endemic countries, where over 50% of TB cases occur in this particularly fragile group. Significant transmission to the native population does not seem to be occurring based on current evidence.^25^–^27^ In low-burden countries TB incidence rates among migrants can be 10–20 times that of the autochthonous population.^5^ Though usually healthy at first arrival in the destination country, such individuals are often latently infected with *M. tuberculosis.* Their extremely marginalized conditions and major changes in lifestyle, leading to immune system weakening, favour the progression of the disease in an active form. Poverty, overcrowded housing, hazardous working environment, and the existence of concomitant risk factors (e.g. illicit drug use, alcohol abuse) are all potential triggers for TB development and spread.^28^ Notably, TB incidence rates among specific migrant communities may differ from what is observed in their country of origin, being either lower or higher as a result of determinants associated to the migration process and the hosting environment. TB re-activation among latently infected subjects more frequently occurs within the first two years after resettlement, probably reflecting their greater psycho-social and economic instability at the beginning of the migratory process.^29^, ^30^

## The Threat of Drug-Resistance

Multi-Drug Resistant TB (MDR-TB) is defined as resistance to, at least, isoniazid and rifampicin among first-line drugs, while Extensively Drug Resistant TB (XDR-TB) refers to MDR-TB with additional resistance to, at least, any fluoroquinolone and any one of three second-line injectable drugs (i.e. capreomycin, kanamycin and amikacin).

**Figure 2:**
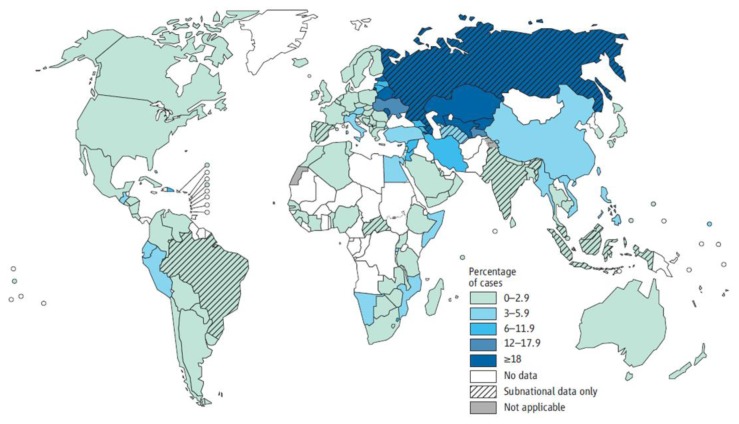
Percentage of new TB cases with multi-drug resistant tuberculosis in 2012 (MDR-TB) [*World Health Organization (WHO), Global Tuberculosis Report 2013*].

MDR-TB prevalence is estimated from the reported proportion of MDR-TB cases in countries that either have a routine drug resistance surveillance system in place or have undertaken a survey and the global TB prevalence.^31^ About 450 000 cases of MDR-TB (range 300 000 – 600 000) were thought to have emerged in 2012, accounting for 3.6% (2.1–5.1%) of all new TB cases and 20.2% (13.3–27.2%) of previously treated cases. The most affected countries are in the Eastern Europe and Central Asia Region (ECA), and include the Russian Federation, Belarus, Azerbaijan, Estonia, Kyrgyzstan, Kazakhstan, Republic of Moldova and Uzbekistan (Figure 2).^1^

Furthermore, XDR-TB, first defined in 2007, has been reported so far by 92 countries in 2012. Overall, it is estimated that 9.6% of all MDR-TB cases are XDR-TB.

The response to MDR-TB has been slow in most countries. Alarmingly, the number of notified MDR-TB cases represents less than 30% of the estimated MDR-TB cases in 2012.^1^ This means that the diagnostic capacity is weak. The implementation of new rapid molecular diagnostic tests will, hopefully, allow to expand testing capacity in low-resource settings worldwide and to improve TB case management. Rapid diagnostic tests such as expert MTB/RIF can be beneficial for both the individual and the community. A significant reduction of the time to diagnosis and treatment for infected patients can lead better outcomes, and so limiting further transmission. In the light of their potential impact on MDR-TB control, the scale-up of such methods should be strongly encouraged, in line with the current WHO recommendations.^32^

With respect to treatment, WHO currently recommends an 8-month intensive phase and a 20-month minimum overall treatment duration.^33^ Second-line drugs show a less favourable toxicity profile, higher costs and more limited accessibility than those included in the standard anti-TB regimen. The report of treatment outcomes for MDR-TB is challenging, with data available for only 62% of the MDR-TB cases notified in the 2010 cohort. Of those, only 48% attained treatment success, which is far from 2015 target of at least 75%.^1^ Operational research projects are currently ongoing in many countries in order to better assess the impact of different second-line regimens.^34^

## TB Control Strategies: Past and Future Steps

After being neglected for two decades, in the early 1990s TB re-emerged in the global health agenda due to outbreaks in high-income countries that prompted renewed attention. In 1993, concerned about the extent of the problem in most of the developing world, WHO declared TB a global emergency.^35^, ^36^ Over the last twenty years global strategies for TB control have been recommended for adoption and adaptation in all countries. The first strategy, DOTS, was launched in 1994–1995. It was based on five key essential components of any sound response to TB: 1) Political commitment with increased and sustained financing; 2) Case detection among people presenting with symptoms in clinics through quality-assured bacteriology; 3) Standardized and supervised treatment along with patient support; 4) Effective drug supply and management system; 5) A standard monitoring and evaluation system [Framework-WHO, 1994, IUATLD, 1996].^37^, ^38^ By adopting this strategy in as many as 180 countries, approximately 17 million patients were started on effective treatment by 2003.^39^

The launch of the first Global Plan to Stop TB 2001–2005 contributed to this achievement by raising prominently the need to invest both domestically and internationally. The establishment of the Global Fund in 2002, by favouring the access to international financing, was an additional strong factor to strengthen national programmes.^40^ As of today, this mechanism has mobilised about 4.6 billion US$ for TB care and control in eligible countries.^41^

In view of the need to accelerate efforts and reach the international targets set in the context of the MDGs, in 2006 WHO launched an enhanced global strategy referred to as Stop TB strategy.^42^, ^43^ This new approach aimed to ensure universal access to high-quality health services and patient-centred care for all individuals with TB, through additional efforts addressing the challenges emerging in the new century.^44^, ^45^ The principles of DOTS were incorporated as the first component of the 2006 Stop-TB Strategy, together with five additional components: 1) address TB/HIV, MDR-TB and the needs of vulnerable populations; 2) contribute to health system strengthening based on primary health care; 3) engage all care providers; 4) empower TB patients and encourage community engagement; 5) enable and promote research.^43^

Underpinned by the new Stop TB Strategy, the second Global Plan to Stop TB covered the period 2006–2015. The scope of this plan was to address the MDG challenge and pursue the other international targets in order to halve the 1990 TB prevalence and mortality rate by 2015 and eliminate TB as a public health problem by 2050 (< 1 case per 1 million population).^46^ Despite all these efforts and the resulting achievements described above, including the reaching of the TB-relevant target in the MDGs, global control is progressing slowly, with a decline in incidence of 2% per year on average. Additional significant challenges remain, such as multidrug-resistance and the limited advances in the search for new drugs, diagnostics, and vaccines.

Nevertheless, in view of recent progress in expansion of proper care and development of new important tools, more ambitious goals and targets have recently been approved by the World Health Assembly in its latest session: Tracing the path towards intensified efforts against TB beyond 2015. The new post-2015 Global TB Strategy approved by the 67^th^ World Health Assembly (WHA) in May 2014, aims at “ending the global TB epidemic” by 2035.^47^ This means a 95% mortality reduction and a 90% incidence decline (< 10 TB cases/100 000 population) by 2035 compared to 2015, and the suppression of any “catastrophic cost” for TB-affected families (Table 1). The strategy stands on three pillars: (1) promote integrated patient-centred care and prevention; (2) foster bold policies and supportive systems; (3) encourage intensified research and innovation (Table 1). As clearly stated in the ten-component list that constitutes the backbone of the post-2015 agenda, a special attention is devoted to the most vulnerable populations (such as migrants, refugees and TB/HIV co-infected patients) and great emphasis is also placed on the need to improve diagnostic and therapeutic capacities through a strong political commitment and intensified investment in research. Crucially, the new strategy recognises fully the need for bold policies that go beyond those of national programmes and engage the entire health system, such as rationalising the use of medicines, ensuring their quality, establishing infection control measures etc. In addition, the new strategy calls upon the institution of universal health coverage and social protection schemes that facilitate access to care by poor people with TB while preventing catastrophic expenditures due to their ill health. Finally, the new strategy emphasises that action on social and economic, “upstream” determinants of TB must be addressed if the world truly envisages the elimination of this disease in the decades to come. Four principles underpin the three pillars: government stewardship and accountability; strong coalition with the civil society and communities; protecting and promoting human rights, ethics and equity; and adaptation of the strategy and targets at country level with global collaboration. In fact, since TB transmission is mainly airborne, and a large submerged reservoir of infection currently exists in the community, global elimination will only be achieved through elimination from every country, which requires international support.

The strategy is also designed to face the challenges of low-burden countries (namely, those with an incidence below 10/100 000/year). In these countries TB tends to concentrate in selected marginalized groups with limited transmission rates within the general population and with most TB cases resulting from reactivation of latent TB infection (LTBI). In an effort to adapt the new strategy to their settings, the 33 countries, that currently can be considered as low-incidence recently, met in Rome for a Global Consultation, convened by WHO and the European Respiratory Society. The topic of their discussions was the common challenges so that they can engage a concerted action to implement the new strategic directives.

Among priorities in these countries, but also worldwide, a crucial importance was attributed to the introduction and the routine application of new technologies for rapid detection of drug-resistance and to the development of specific diagnostic algorithms, particularly useful for high-risk patients.^48^

After over 40 years from the development of rifampicin in the 60s, two new anti-tuberculosis drugs were registered in 2012 and 2013. Bedaquiline was approved by the US Food and Drug Administration (FDA) at the end of 2012 and is currently recommended by WHO for the treatment of selected MDR-TB cases.^49^ A year later, Delamanid received the approval from the European Medical Agency (EMA) and Bedaquiline is currently available for use in MDR-TB treatment in Europe.^50^ Currentely, the pharmaceutical industry has little interests in investing on research and manufacturing new anti-tubercular molecules that at present are considered orphan drugs. A cautious choice of patients and close monitoring during and after treatment are, therefore, essential to preserving their efficacy.

## Figures and Tables

**Table 1:** The post-2015 global TB strategy [adapted from WHO, *Global strategy and targets for tuberculosis prevention, care and control beyond 2015*, http://www.who.int/entity/tb/post2015_TBstrategy.pdf?ua=1]

**VISION**	A world free of tuberculosis –zero deaths, disease and suffering due to tuberculosis
**GOAL**	**End the global tuberculosis epidemic**
**MILESTONES FOR 2025**	–75% reduction in tuberculosis deaths (compared with 2015); –50% reduction in tuberculosis incidence rate (compared with 2015) (less than 55 tuberculosis cases per 100 000 population) –No affected families facing catastrophic costs due to tuberculosis
**TARGETS FOR 2035**	–95% reduction in tuberculosis deaths (compared with 2015) –90% reduction in tuberculosis incidence rate (compared with 2015) (less than 10 tuberculosis cases per 100 000 population) –No affected families facing catastrophic costs due to tuberculosis
